# Isolated Pulmonary Valve Endocarditis Caused by *Enterococcus faecalis*—Facing the Unpredictable

**DOI:** 10.3390/antibiotics14030220

**Published:** 2025-02-21

**Authors:** Andrei Vâţă, Isabela Ioana Loghin, Radu Ștefan Miftode, Daniela Crişu, Cătălina Mihaela Luca, Alin Mihai Vasilescu, Ioana Maria Onofrei, Tudorița Parângă, Ionela-Larisa Miftode

**Affiliations:** 1 Department of Infectious Diseases, “Grigore T. Popa” University of Medicine and Pharmacy, 700115 Iași, Romania; andrei.vata@umfiasi.ro (A.V.); catalina.luca@umfiasi.ro (C.M.L.); maria-ioana.hunea@umfiasi.ro (I.M.O.); tudorita.paranga@umfiasi.ro (T.P.); ionela-larisa.miftode@umfiasi.ro (I.-L.M.); 2 “St Parascheva” Clinical Hospital of Infectious Diseases, 700116 Iași, Romania; 3 Department of Cardiology, “Grigore T. Popa” University of Medicine and Pharmacy, 700115 Iași, Romania; radu-stefan.miftode@yahoo.com (R.Ș.M.);; 4 “St Spiridon” Emergency Hospital, 700115 Iași, Romania; alin.vasilescu@umfiasi.ro; 5 Department of General Surgery, “Grigore T. Popa” University of Medicine and Pharmacy, 700115 Iași, Romania

**Keywords:** infective endocarditis, *Enterococcus faecalis*, antibiotic resistance

## Abstract

**Background:** Infective endocarditis is a severe, life-threatening condition which affects the endocardial lining of the heart. Right-sided IE typically affects the tricuspid valve and, less commonly, the pulmonary valve, often in association with intravenous drug use and intracardiac devices. *Enterococcus faecalis*, a significant pathogen in healthcare settings, is frequently resistant to several antibiotics, complicating treatment. **Results:** We present the case of a 69-year-old man diagnosed with isolated pulmonary valve endocarditis, with no identifiable risk factors, good oral hygiene, and no recent dental procedures. In our case, the *E. faecalis* strain, initially susceptible to ampicillin, acquired resistance during high-dose ampicillin and gentamicin therapy, leading to cardiac surgery and prolonged antibiotic treatment. **Conclusions:** IPE is rare, emphasizing the need for heightened clinical awareness and thorough diagnostic evaluation, especially in patients with endocarditis symptoms and known risk factors. Although ampicillin resistance in *E. faecalis* is uncommon, its emergence during treatment presents a challenge, necessitating careful antibiotic stewardship to improve outcomes.

## 1. Introduction

Infective endocarditis (IE) is a serious infection involving the endocardial lining of the heart, typically caused by various bacterial pathogens. In recent years, the epidemiology of IE has undergone significant changes, influenced by evolving risk factors, an increase in invasive medical procedures, and a rise in antibiotic-resistant organisms. These shifts have impacted the prevalence, patient demographics, and outcomes associated with IE, necessitating updated approaches to its prevention, diagnosis, and treatment [1].

A recent study that analyzed data from several European countries showed a twofold increase in the incidence of IE in Europe between 2000 and 2018 [1]. This increase in IE incidence was probably a result of multiple factors working together, including the growing number of older people, who are generally more susceptible to infections, more people with implanted cardiovascular devices (such as pacemakers or artificial valves), the use of a wider variety of imaging techniques to diagnose IE, which might lead to more cases being identified, improvements in medical coding practices, which could also contribute to a perceived increase in cases, and maybe also the limitations regarding the use of antibiotics for prophylaxis (prevention) [1]. In 2019, the estimated European age-standardized incidence rate of IE was 13.80 per 100,000 person-years [2].

Right-sided infective endocarditis (RSIE) is a relatively rare form of IE, accounting for approximately 5–10% of all IE cases. RSIE primarily usually involves the tricuspid valve and, less frequently, the pulmonary valve. While the epidemiology of left-sided endocarditis is more commonly associated with structural heart disease and prosthetic valves, RSIE has a unique set of risk factors, particularly linked to IVDU, the presence of intracardiac devices, long-term catheter use, and certain congenital heart conditions [3]

*Enterococcus* species, particularly *Enterococcus faecalis* and *Enterococcus faecium*, are important causes of infective endocarditis. They rank as the third most common etiological agents of this serious infection, following staphylococci and streptococci, and are responsible for approximately 10% to 15% of all endocarditis cases [4,5,6]. The virulence of these organisms is attributed to several factors, including their ability to form biofilms, which are critical for their adherence to heart valves and subsequent colonization [7,8]. *E. faecalis* is the predominant species involved in enterococcal endocarditis, accounting for about 90% of cases, while *E. faecium* contributes to a smaller percentage [5,9]. *Enterococcus faecalis* is a significant pathogen in healthcare settings. This bacterium is a common cause of nosocomial infections, including urinary tract infections, endocarditis, and bacteremia [10]. The rise of multidrug-resistant *E. faecalis* strains is a significant public health concern, as these strains can lead to treatment failures and increased morbidity and mortality among infected patients. *E. faecalis* has an intrinsic resistance to numerous antibiotics, including fusidic acid, cephalosporins (including ceftazidime), macrolides, clindamycin, sulfonamides, and quinupristin-dalfopristin [11]. Its standard treatment often involves the combination of ampicillin with aminoglycosides; however, the emergence of high-level resistance to aminoglycosides could further complicate therapeutic strategies [12]. Currently, one of the primary concerns regarding *E. faecalis* is its resistance to vancomycin. The prevalence of vancomycin-resistant *E. faecalis* (VRE) has been rising, particularly in Europe, where regional differences in resistance rates have been documented; countries like Greece, the UK, and Portugal report higher incidences of vancomycin resistance compared to other regions [10,13]. The mechanisms of resistance include the acquisition of the vanA gene, which alters the target site of vancomycin, rendering it ineffective [10].

When evaluating a patient with prolonged fever, it is crucial to consider a wide range of differential diagnostics, including the possibility of IE. A delayed IE diagnosis can result in an array of serious complications due to the ongoing infectious process and structural injury, both cardiac and systemic. The prompt recognition and subsequent management of IE are of paramount importance for the prevention of these potentially life-threatening outcomes. The overall in-hospital mortality rate for IE ranges from 15% to 25% and its 1-year mortality rate has been as high as 40% in some studies, reflecting the severity of this disease and its complications [14,15,16]. The mortality rates associated with IE in Europe have shown a decline in recent years, likely due to improved diagnostic and therapeutic approaches. However, they remain high, particularly in patients with severe infections or underlying comorbidities [17]. Surgery, although frequently indicated, is rejected in 24–69% of cases because of the procedure’s risks. In-hospital mortality is significant after surgery (29–50%), but highest in patients rejected for operation (52–83%) [18].

Our objective is to present and discuss a rare case of isolated pulmonary valve infective endocarditis caused by a strain of *Enterococcus faecalis* that probably developed multidrug resistance (MDR) during antibiotic treatment. This resistance ultimately led to the necessity of surgical valve replacement to effectively manage the infection.

## 2. The Case

We report the case of a 69-year-old man living in a rural area who was admitted to the hospital with symptoms including chills, fever (peaking at 38.6 °C), headache, dizziness, pronounced physical weakness, polyuria, nocturia, loss of appetite, and significant weight loss (approximately 10 kg over three weeks). His medical history included essential hypertension, managed with amlodipine and bisoprolol, chronic prostatitis treated with tamsulosin and dutasteride, and chronic hepatitis B, for which he had been on long-term entecavir therapy. He reported no history of smoking, alcohol use, or intravenous drug use.

The symptoms had developed progressively in the previous 14 days before admission, with intermittent fever, accompanied by chills and the exacerbation of chronic urinary symptoms. The family physician initially recommended urinalysis and treatment with oral levofloxacin, which lasted 10 days and resulted in partial remission of the fever, but no improvement in appetite or general condition. The urine culture came back negative. After the completion of the antibiotic regimen, the reoccurrence of fever and chills was noted. A thoracic, abdominal, and pelvic contrast-enhanced CT scan was performed, with no signs of acute infection or abscesses. Another urine culture showed no pathological results. An oral course of amoxicillin-clavulanate was initiated, with little to no improvement.

Upon admission to the hospital, the patient presented with a fever of 38.0 °C and diaphoresis. His blood pressure was 115/70 mm Hg, and his heart rate was 92 beats per minute. A faint systolic murmur was noted in the left second intercostal space. Physical examination revealed moderate hepatomegaly and a mildly enlarged spleen, although the patient reported no abdominal pain. There was no peripheral edema or other indications of cardiac decompensation.

The initial laboratory findings revealed leukocytosis (22,750 WBC/mm^3^) with an elevated neutrophil count (19,565/mm^3^), high C-reactive protein (CRP) at 97.1 mg/L, and an erythrocyte sedimentation rate (ESR) of 60 mm/h. The patient had mild normocytic anemia (Hb 11.2 g/dL). His renal function was normal; no hepatic cytolysis was present.

**Figure 1:**
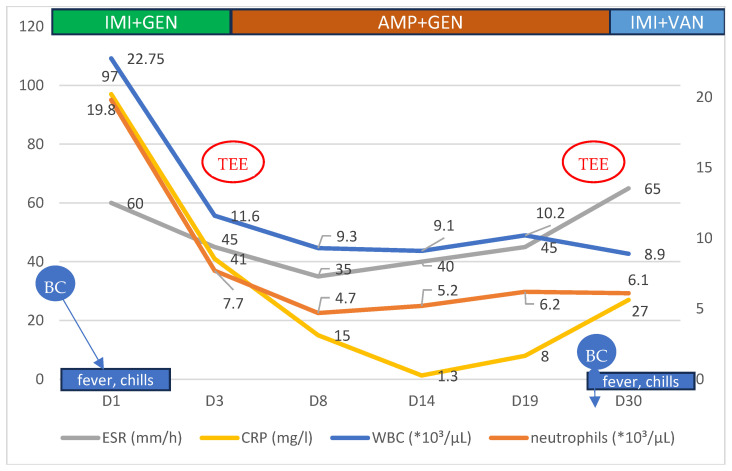
Evolution of some of the hematological and biochemical parameters of the patient, according to symptoms and treatment regimens during the hospital stay. BC—blood culture, TEE—transesophageal echocardiography, IMI—imipenem, GEN—gentamicin, AMP—ampicillin, and VAN—vancomycin.

After a 24 h pause in antibiotic treatment, during which multiple blood cultures were obtained and sent for microbiological analysis, an empirical antibiotic regimen of imipenem/cilastatin (1 g twice daily) and gentamicin (240 mg once daily) was initiated. The patient showed a favorable response, with the resolution of fever and reductions in both his leukocyte count and inflammatory markers (Figure 1).

**Figure 2:**
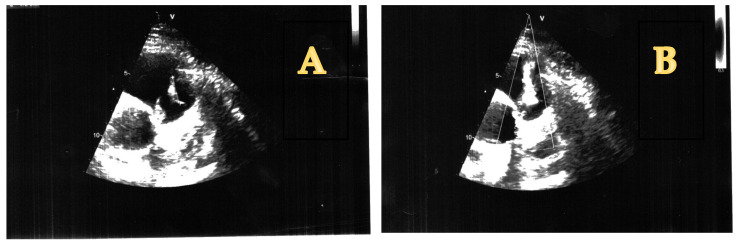
(**A**) Pulmonary valve vegetations (short axis view)**.** (**B**) Pulmonary valve regurgitation caused by EI (short axis view).

On the 5th day after admission, a transthoracic echocardiography was performed, which showed the presence of two vegetations at the level of the pulmonary valve, as follows: 21 mm on the posterior cusp and 17 mm on the anterior cusp, determining a moderate-to-severe pulmonary regurgitation, with a maximum gradient of 12 mm Hg between the right ventricle and pulmonary artery. A moderate tricuspid regurgitation with an eccentric flow towards the interatrial septum was recorded as well, with a maximum gradient of 40 mm Hg. However, the IVC was not dilated, with a normal inspiratory collapse. The right ventricle had a normal size and systolic function (tricuspid annular planes excursion of 30 mm). The mitral and aortic valves were structurally normal. The left ventricle had a normal size and systolic function, with a left ventricular ejection fraction calculated at 60% with the Simpson method (Figure 2).

An oral cavity examination was performed, but no cavities or other significant morphological issues were found; there were no periapical interventions, colonoscopy or colitis episodes, or i.v. treatments in the 3-month period before the onset of symptoms. We could not identify any skin abrasions or lesions that could act as an entry gate for the pathogen; he strongly denied the use of any i.v. or other type of drugs. The patient was advised to undergo a colonoscopy, but declined the recommendation.

On the 6th day from the blood cultures drawn on day 1, a strain of *Enterococcus faecalis* susceptible to ampicillin with an intrinsic low-level resistance to gentamicin was isolated (the antibiotic susceptibility testing was performed using the disk diffusion method, following the EUCAST clinical breakpoints guidelines).

Subsequently, the antibiotic treatment was changed to ampicillin at 3 g q.i.d. and gentamicin at 240 mg q.d. After 14 days, the dose of gentamicin was lowered to 160 mg q.d. for another 10 days. The clinical evolution was favorable, with no fever or chills and an improvement in the patient’s general condition and appetite.

On day 27 of treatment, the patient experienced a recurrence of chills, fever, and sweating, despite being on a high-dose regimen of ampicillin. Laboratory tests showed an elevated erythrocyte sedimentation rate (ESR), while the leukocyte count remained within the normal range.

**Figure 3:**
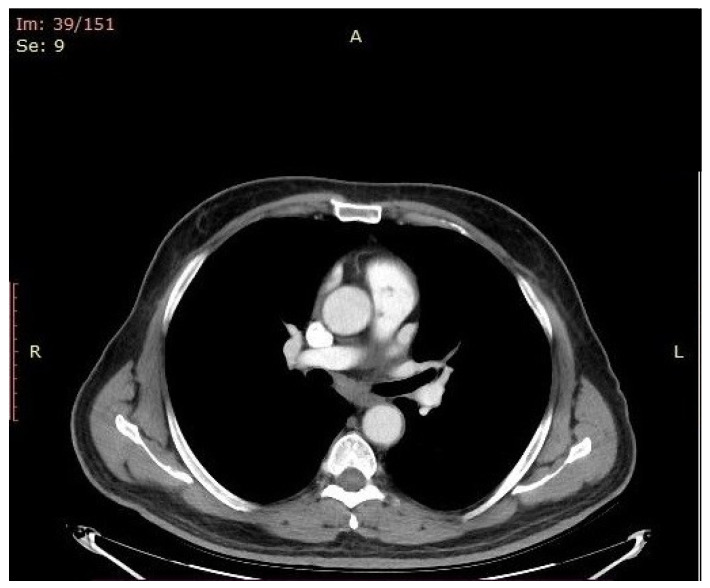
Pulmonary valve vegetations as depicted in CT scan images.

The antibiotic was withheld again (for 24 h) and new sets of blood and urine cultures were drawn. A new TEE and CT scan (Figure 3) were performed, and no improvement was observed in the size or number of the pulmonary valve vegetations.

The antibiotic treatment was re-initiated with vancomycin (1 g bid) and imipenem-cilastatin (1 g bid), with little effect on the patient’s symptoms. After another 6 days, a strain of *Enterococcus faecalis* resistant to ampicillin, vancomycin, and teicoplanin was isolated from the blood cultures. The only antibiotic to which the bacterium was susceptible was linezolid.

Given the clinical failure of antibiotic therapy and the persistence of vegetations, we consulted with multiple local cardiovascular surgeons. The patient was subsequently referred to Monza Hospital in Bucharest, where he underwent pulmonary valve replacement via sternotomy using a Magna Ease no. 25 prosthetic valve. Cultures from the extracted vegetations and post-operative blood samples showed no bacterial growth.

**Figure 4:**
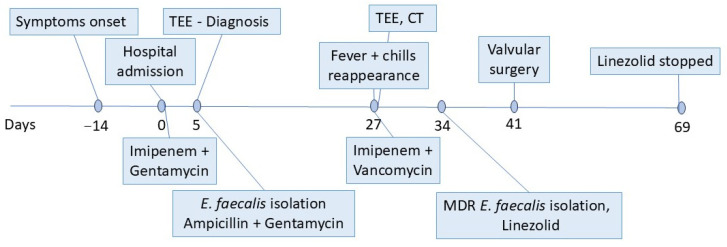
Timeline representing the patient’s diagnostic and therapeutic interventions. TEE—transesophageal echocardiography; CT—computed tomography; and MDR—multidrug resistant.

Following surgery, the patient completed a four-week course of linezolid (600 mg twice daily), and his post-surgical recovery was uneventful (Figure 4). Clinical, biological, and ultrasound evaluations conducted at 1, 3, 6, and 12 months after surgery revealed no symptoms or significant abnormalities, indicating a stable recovery.

## 3. Discussion

Isolated pulmonary valve endocarditis (IPE) is a rare form of infective endocarditis, accounting for approximately 1.5% to 2% of all endocarditis cases reported in various studies [19,20]. This low incidence of IPE can be attributed to several factors, including the hemodynamic characteristics of the right side of the heart, which operates under a lower pressure compared to the left side of the heart, and the relatively lower prevalence of congenital or acquired valvular abnormalities affecting the pulmonary valve [19,21,22].

The clinical presentation of IPE often includes respiratory symptoms due to the anatomical proximity of the pulmonary valve to the lungs, which can lead to complications such as septic emboli affecting the pulmonary circulation [23,24]. Our patient’s symptoms were dominated by fever, chills, and fatigue, an association frequently seen in IE; his loss of appetite that led to an significant weight loss was somehow unusual, but no other causes were identified. He had no pulmonary symptoms at onset or during hospitalization.

Despite its rarity, IPE can occur in various patient populations, with underlying risk factors including those with congenital heart defects, intravenous drug users, and patients undergoing procedures like transcatheter pulmonary valve implantation or those with the presence of central venous catheters [19,20]. We could not identify any of those risk factors in our patient; he had a good oral hygiene and no recent dental interventions. Chronic prostatitis, as in our patient, can be a source for *E. faecalis* bacteremia [25]. However, in this case, the source remained uncertain, as his urine cultures were negative and the patient declined the recommended colonoscopy.

The emergence of bacterial resistance to antibiotics is often linked to the overuse and misuse of antibiotics in both healthcare and agricultural settings. In our case, we had to deal with an *E. faecalis* strain initially susceptible to ampicillin, which probably acquired resistance during therapy with a high-dose ampicillin and gentamicin regimen. The emergence of ampicillin resistance in *E. faecalis* during treatment is a growing concern in clinical microbiology [10]. Although it is generally less resistant to ampicillin compared to its counterpart, *Enterococcus faecium*, there have been documented instances of resistance development, particularly in nosocomial settings [26]. Our patients’ IE was caused by an initially ampicillin-susceptible *E. faecalis* strain (isolated from several blood cultures before the start of the antibiotic therapy). It seems to have developed ampicillin and vancomycin resistance during therapy (with high i.v. doses of ampicillin, as per the ESC guidelines’ recommendations [27]) (Table 1).

Another explanation for the MDR *E. faecalis* strain isolated after high-dose ampicillin treatment could be a re-infection of the previously damaged endocardium of the pulmonary valve from a persistent primary site of infection (maybe prostatic). We did not have access to molecular or phenotypic methods to identify strain-specific characteristics and differentiate the two isolates.

This *E. faecalis* strain acquiring antibiotic resistance can be attributed to various factors, including genetic mutations, the selective pressure exerted by antibiotic treatment, and the intrinsic characteristics of the bacteria. A study reported that the prevalence of ampicillin-resistant *E. faecalis* isolates was approximately 3.6% in clinical settings, which is consistent with findings from other regions where resistance rates are typically low [28]. However, there are reports of increased resistance rates in specific geographical areas, therefore, local epidemiological factors and antibiotic usage patterns can influence resistance emergence [29]. The mechanisms underlying ampicillin resistance in *E. faecalis* primarily involve alterations in penicillin binding proteins (PBPs), which are critical for the antibiotic’s efficacy. Mutations in or the overproduction of low-affinity PBPs can lead to a reduced binding of ampicillin, thereby conferring resistance [30]. Additionally, the presence of β-lactamase enzymes, although less common in *E. faecalis* compared to *E. faecium*, can also contribute to resistance by hydrolyzing the antibiotic [30]. Moreover, selective pressure from ampicillin treatment can lead to the selection of resistant strains.

In a clinical context, prolonged or inappropriate courses of ampicillin may inadvertently promote the survival and proliferation of resistant *E. faecalis* strains [31]. In our case, we used high-dose ampicillin at 3 g every 6 h, and our records show no deviation from this schedule.

The implications of ampicillin resistance in *E. faecalis* are significant, as it limits treatment options and complicates the management of enterococcal infections. As such, clinicians must remain vigilant in monitoring resistance patterns and consider alternative treatment regimens [32].

Despite the fact that, in most cases, the first and most used therapeutic approach in IE is antibiotic treatment, in some patients, cardiac surgery in the acute phase can offer up to a 20% survival advantage in the first year [33,34]. The indications for surgery have evolved in the last years, and according to the latest ESC guidelines [27], they include heart failure and a high risk of or an established embolism or uncontrolled infection (witnessed by local complications (abscess, false aneurysm, fistula, and enlarging vegetation), persistent positive blood cultures, or resistant bacteria or fungi—*S. aureus* (methicillin resistant), vancomycin-resistant enterococci, and non-HACEK Gram-negative bacteria).

Our patient had several indications for surgical intervention, as follows: persistent positive blood cultures after 4 weeks of antibiotic treatment, no improvement in the size of vegetations, and the emergence of antibiotic resistance in the isolated *E. faecalis*. The cardiovascular surgeon opted for the Carpentier-Edwards Perimount Magna Ease valve—a bioprosthetic option used in pulmonary valve replacement, particularly during sternotomy procedures. This valve is designed to provide an improved hemodynamic performance and durability compared to other prosthetic options [35] and has demonstrated satisfactory outcomes in terms of mortality and valve-related complications over extended follow-up periods [36]; it does not require long-term anticoagulation [37].

The moment of the surgical intervention is important for obtaining the best results in patients with IE, and there are still debates among specialists about this subject [38,39,40]. Our patient could have had an opportunity for earlier surgery if we would have repeated his blood cultures sooner, but his initial favorable clinical and inflammatory markers evolution misled us.

The importance of careful postoperative monitoring and management cannot be overstated, as it plays a significant role in the long-term success of valve replacements. Moreover, peripheral embolism and infective-endocarditis-associated heart failure independently correlate with major adverse events and all-cause death, highlighting the critical role of embolic risk in determining the need for early surgical intervention [41]. Our patient was followed up by us for one year after surgery and showed a good recovery, with no new signs or symptoms suggestive of a new episode of IE or cardiac impairment.

## 4. Conclusions

The rarity of isolated pulmonary valve endocarditis underscores the importance of increased clinical vigilance and comprehensive diagnostic assessment in patients with symptoms indicative of endocarditis, especially among those with known predisposing factors. Changes in the antimicrobial resistance of *Enterococcus faecalis* during treatment pose a significant challenge in clinical practice, potentially arising from an uncontrolled intestinal source or the in vivo selection of resistant strains. Addressing this issue requires on-going surveillance and carefully tailored antibiotic stewardship to reduce the impact of resistance and maintain the effectiveness of treatments for enterococcal infections. Cardiac surgery in the acute phase of IE can significantly improve outcomes, but it remains a high-risk procedure, and careful consideration of the patient’s clinical condition and the extent of the disease is essential in decision making.

## Figures and Tables

**Table 1:** Antibiotic susceptibility and MIC breakpoints of the two E. faecalis isolates (on day 1 and day 28).

Antibiotic Tested	Susceptibility		MIC Breakpoints (mg/L)	
	First Isolate	Second Isolate	First Isolate	Second Isolate
Ampicillin	S	R	ND *	16
Gentamicin	LR	HR	128	512
Vancomycin	S	R	1	16
Linezolid	S	S	2	2

MIC—minimum inhibitory concentration; ND—not done. S—susceptible, R—resistant, LR—low resistance, HR—high resistance * As per Eucast guidelines, only *E. faecalis* that test resistant to ampicillin with disk diffusion should be confirmed with and MIC test.

## Data Availability

Data are contained within the article.

## Abbreviations

The following abbreviations are used in this manuscript:

IPE	Isolated pulmonary valve endocarditis
IE	Infective endocarditis
VRE	Vancomycin resistant *E. faecalis*
IVDU	Intravenous drug use
MDR	Multidrug resistance
CRP	C-reactive protein
BC	Blood cultures
GEN	Gentamicin
IMI	Imipenem
AMP	Ampicillin
VAN	Vancomycin
TEE	Transesophageal echocardiography
ESR	Erythrocyte sedimentation rate
RSIE	Right-sided infective endocarditis
PBP	Penicillin binding protein
Hb	Hemoglobin
